# Genotypic characterization of amoeba isolated from *Acanthamoeba* keratitis in Poland

**DOI:** 10.1007/s00436-015-4319-0

**Published:** 2015-01-22

**Authors:** Monika Derda, Piotr Solarczyk, Marcin Cholewiński, Edward Hadaś

**Affiliations:** Department of Biology and Medical Parasitology, Poznan University of Medical Sciences, Poznan, Poland

**Keywords:** *Acanthamoeba*, *Acanthamoeba* keratitis, Cornea, Genotyping

## Abstract

Free-living amoebae belonging to the genus *Acanthamoeba* are the causative factor of many diseases. Among others, they cause *Acanthamoeba* keratitis (AK), a condition that usually occurs in contact lens wearers, though it is also observed in non-wearers. The number of diagnosed cases of AK increased more than eightfold during 8 years in the USA, and a proportional increase in frequency also occurred in Poland and Europe. Cases of AK are usually diagnosed late, and their therapy is difficult and rarely successful. AK is an uncommon diagnosis in Poland. The increased number of positive cases observed in our laboratory may reflect the growing at-risk population of contact lens wearers. *Acanthamoeba* as a genus of facultative human parasites is currently classified into 17 genotypes. Isolates belonging to seven genotypes were found to be associated with AK. One genotype in particular, T4, was found to be overrepresented in human disease. The main finding of our study is that in Poland, AK is almost always associated with the T4 genotype.

## Introduction

Amoebae belonging to the genus *Acanthamoeba* are free-living organisms, widespread in the environment, and excellently adjusted to various environmental conditions (Stockman et al. 2011). They occur in two developmental stages: trophozoites and cysts, resistant to environmental conditions (Marciano-Cabral and Cabral 2003). The invasive forms for the human are cysts as well as trophozoites of amoebae. Infection occurs most often in the warm seasons and is associated with contact with water. In particular cases, the infection may take place through damaged skin or accidently injured cornea, e.g., during earth works or playing with sand or water, or through contact lenses.

The most significant and most common disease caused by the free-living amoebae is *Acanthamoeba* keratitis (AK).

The first cases of the amoeba’s invasion of the cornea were described in 1974 (Naginton et al. 1974). In subsequent years, only a few cases of AK were described. After 1981, a rapid increase in the number of cases of keratitis was observed, which reached a peak in 1985. Most cases were described in the USA.

Within the last two decades, amoebae of the *Acanthamoeba* genus have become a well-known and significant risk factor for the keratitis. Contributory factors include global climate warming and the increase in the population of patients with immunological deficiencies. The number of diagnosed cases of AK in the USA increased from 22 in 1999 to 170 in 2007 (Yoder et al. 2012). A proportional increase of the number of diagnosed cases of AK also occurred in Poland.

So far, apart from the USA, AK cases have been described in Europe, Australia, Asia, and Africa. The approximate number of infections is counted in thousands. The majority of cases, as many as 85–88 %, are connected with the wearing of contact lenses (Dart et al. 2009). Among this group, 88 % of cases are connected with wearing hydrogel contact lenses and 12 % with wearing rigid lenses. Seal (2003) stated that the probability of contracting AK each year is 1:30,000 in persons wearing contact lenses.

In persons not wearing contact lenses, AK is usually diagnosed late, and in these cases, the disease is usually already at an advanced stage.

AK usually affects one eye. The first symptoms are blurred vision, photophobia, and severe pain in the eye not explained by corneal injury. Conjunctival swelling and eyelid swelling also appear. Patients have bleary and teary eyes. In the front layers of the corneal stroma, there are diffuse, ring-shaped, or crescent infiltrates and less specific satellite infiltrates. The ring-shaped infiltrates are single or numerous. The epithelium covering the corneal stroma can be unharmed or may have punctate erosions. In the late stage of the disease, pus appears in the eye. Together with the development of the disease, the corneal edema increases (Kosik-Bogacka et al. 2010).

## Material and methods

### Samples of amoebae

Eight *Acanthamoeba* strains were isolated from corneal scrapings from patients suspected of having keratitis. Scrapings were placed on 2 % non-nutrient (NN) agar plates, covered with *Enterobacter aerogenes* bacteria, and the plates were incubated at a temperature of 28 °C. After 3–5 days, the increase in the number of amoebae was observed and examined with an inverted microscope at × 200. The plates were monitored microscopically for up to 2 weeks for growth of *Acanthamoeba* trophozoites or for the presence of cysts.

### Pathogenicity test

The amoebae 2–3 days old obtained from the NN agar culture were washed down with sterile distilled water. The suspension thus obtained was thickened and used to infect 2-week-old mice, strain BALB/c, by intranasal inoculation of five mice for each isolate. The mice were kept for 2 weeks, and thereafter anaesthetized and killed, unless they died within the first few days of being infected. The brains and lungs of the mice, irrespective of the method of death, were collected in order to isolate the amoebae. This study was conducted to verify the pathogenicity and virulence of isolated amoeba.

### Molecular identification

The DNA amplification was performed using genus-specific primers previously described by Schroeder et al. (2001). A set of primers that included the forward JDPI (5′GGCCCAGATCGTTTACCGTGAA′3), and the reverse primer JDP2 were used (5′TCTCACAAGCTGCTAGGGAGTCA′3) for genetic characterization targeting the ~450 bp fragment of the *Acanthamoeba* 18S ribosomal rRNA (rRNA) gene. Amplification involved use of a 25 μl suspension of the following reagents: 2.5 mM MgCl_2_, 0.6–1 μM of each primer, 0.2 mM of each deoxynucleotide triphosphate, and 0.5 U of AmpliTaq Gold DNA polymerase. A clinical isolate of *Acanthamoeba castellanii* belonging to the T4 genotype isolated from a keratitis patient (ATCC 50374) was used as a positive control. A negative control consisting of a reaction mixture without a DNA template was included. Polymerase chain reaction (PCR) was carried out using a GeneAmp 2400 thermocycler. PCR products were analyzed on 1 % agarose gel stained with ethidium bromide. Gel images were illuminated using UV light and captured using a gel documentation system. PCR products were cleaned and sequenced in both directions with the same set of primers. Sequencing was performed with BigDye Terminator v3.1 on an ABI Prism 3130XL Analyzer (Applied Biosystems, USA). Trace files were checked and edited using FinchTV 1.3.1 (Geospiza Inc., Seattle, USA). Contigs were aligned and manually assembled in GeneDoc v. 2.7.000 (Nicholas and Nicholas Jr 1997; Nicholas et al. 1997). Sequencing was conducted in both directions with the same set of primers. Sequences were analyzed using the program Chromas. Next, the gene sequence fragments of the *Acanthamoeba* isolates were compared with the reference sequences deposited in GenBank (National Center for Biotechnology Information).

## Results

### Pathogenicity test

All studied isolates of amoebae were highly pathogenic and virulent. Among five infected mice, five died within 7–10 days, and the amoebae were isolated from them again.

### Molecular identification

The study results of genotyping of amoebae isolates from human cases of AK from Poland are presented in Table 1. The obtained nucleotide sequences were compared with the sequences previously determined and deposited in GenBank.

**Table 1:** Comparison of strains isolated from human cases of AK compared to reference strains deposited in GenBank

Strains isolated from AK cases in Poland		100 % nucleotide sequence similarity in relation to reference strains			
Strain	Accession no.	Origin	Region of origin	Accession no.	Reference
Ac32 Ac35 Ac36 Ac37	KP184479 KP184481 KP184482 KP184483	*Acanthamoeba* sp., AcaVN15, dialysis unit water	Slovakia	GQ397477	Nagyova et al. 2010a
		*Acanthamoeba* sp., AcaVN14, salt cave scrape	Slovakia	GQ397476	Nagyova et al. 2010a
		*Acanthamoeba* sp., CRIB-23, river water—Seine River	France	EU273825	Thomas et al. 2008
Ac34 Ac48 Ac55	KP184480 KP184485 KP120880	*Acanthamoeba* sp., CDCV600, liver of a Temminck's tragopan	USA	GQ889265	Visvesvara et al. 2010
		*Acanthamoeba* sp., S6, contact lens and contact lens case	France	DQ087296	Yera et al. 2008
		*Acanthamoeba* sp., CCS-LG, keratitis,	Brazil	JX441874	Duarte et al. 2013
		*Acanthamoeba* sp., 1 FRC-2013, corneal surface tissue	Brazil	KF318460	Mafra et al. 2013
		*Acanthamoeba* sp., CRIB53, biofilm	France	EU377583	Corsaro et al. 2009
		*Acanthamoeba* sp. 222BAL corneal scraping	France	DQ087297	Yera et al. 2007
Ac46	KP184484	*Acanthamoeba* sp., AR881, swamp water	Spain	JQ678632	Garcia et al. 2013
		*Acanthamoeba* sp., AcaVNAK03, corneal scrape	Slovakia	GQ905497	Nagyova et al. 2010b

It was found that all obtained sequences of amoebae isolates from the cases of AK belong to the genotype T4 *Acanthamoeba* sp. and were 100 % identical to the sequences of the same marker obtained from environmental samples but also from people. It was also found that there are small deviations from the typical model, revealing a certain polymorphism within genotype T4. It was found that the particular amoebae isolates form three groups of nucleotide sequences are 100 % identical to the model strains deposited in GenBank.

It was found that the DNA sequences of the fragment of the gene *18S rRNA* Ac34, Ac48, and Ac55 are 100 % identical to reference sequences of the isolates of *Acanthamoeba* strain S6 [accession no. in GenBank DQ087296] (Yera et al. 2008), strain CCS-LG [JX441874] (Duarte et al. 2013), strain 1 FRC-2013 [KF318460] (Mafra et al. 2013), strain CDCV600 [GQ889265] (Visvesvara et al. 2010), strain CRIB53 [EU377583] (Corsaro et al. 2009), and strain 222BAL [DQ087297] (Yera et al. 2007), originating from Brazil, the USA, and France, isolated from human cases of AK (Yera et al. 2008; Duarte et al. 2013; Mafra et al. 2013; Yera et al. 2007) as well as infected liver of Temminck’s tragopan (Visvesvara et al. 2010) and the environment (Corsario et al. 2009).

The compared sequences of the Ac32, Ac35, Ac36, and Ac37 isolates from AK show 100 % similarity to the sequences of the same marker obtained from environmental samples, identified as *Acanthamoeba* sp. of genotype T4 of such isolates as: strain AcaVN15 from dialysis unit water [GQ397477], strain AcaVN14 from salt cave scrape [GQ397476] (Nagyova et al. 2010a), and strain CRIB-23 from river water [EU273825]. The isolate Ac46 was identical in terms of nucleotide sequences to strain AR881 from swamp water [JQ678632] (Garcia et al. 2013) and strain AcaVNAK03 from corneal scrape [GQ905497] (Nagyova et al. 2010b).

Our *Acanthamoeba* sequences from the Ac32, Ac34, Ac35, Ac36, Ac37, Ac47, and Ac48 isolates obtained from corneal scrapings were deposited in GenBank (NCBI) under accession numbers KP184479 to KP184485.

## Discussion

The omnipresence of amoeba in the surrounding environment is undeniable. These cosmopolitan organisms are found in samples of soil, air, water, and animal tissues. *Acanthamoeba* spp. have been isolated and identified in many countries around the world, for example in Bulgaria (Tsetkova et al. 2004), Iran (Mahmoudi et al. 2012), Spain (Magnet et al. 2012), Brazil (Duarte et al. 2013), Japan (Edagawa et al. 2009), the USA (John and Howard 1995), Switzerland (Gianinazzi et al. 2009), and also in Poland (Kasprzak and Mazur 1972).

Recognition of invading amoeba of the genus *Acanthamoeba* in the eye is difficult. Numerous corneal infections caused by viruses, fungi, or bacteria cause symptoms similar to amoeba infection. Very often, an infection of the eye is mistakenly diagnosed as an infection caused by herpes simplex viruses.

In the case of inflammation of the cornea caused by *Acanthamoeba*, there are no uniform standards for diagnostic procedures. The most common diagnostic method is culture of corneal scrapings on NN agar coated with bacteria (Borin et al. 2013). Methods of visualization of the cornea using confocal microscopy and immunofluorescence are also used (Aniśko-Słomińska et al. 2011; Kokot et al. 2012). Additional methods of detection include molecular analysis of nuclear and mitochondrial DNA and methods of testing restriction fragment length polymorphism analysis after digestion of DNA (Kosik-Bogacka et al. 2010).

The taxonomy of the small amoeba species is not well established. Consequently, little is known about the genetic relationships between pathogenic and nonpathogenic strains of *Acanthamoeba*. During the last few years, molecular techniques (such as polymerase chain reaction—PCR) are increasingly being used in genotype identification (Booton et al. 2005; Caumo et al. 2009; Goldschmidt et al. 2012; Howe et al. 1997; Itahashi et al. 2011; Jain and Tilak 2011; Laummaunwai et al. 2012; Le Calvez et al. 2012). Our previous study confirmed the usefulness of PCR for the early, rapid, and sensitive diagnosis of pathogenic *Acanthamoeba* spp. strains (Derda et al. 2014). The best highly specific marker that distinguishes pathogenic *Acanthamoeba* strains in water samples in Poland is a pair of primers of Aca 16. The first results confirming the sensitivity and specificity of a PCR assay for the detection of AK were demonstrated by Lehmann et al. (1998), using two different pairs of primers (P1GP, P2GP). An interesting study showed that a combination of primer pairs (ACARNA, JDP, and NELSON) in the PCR-based method for diagnosing AK is more sensitive than culture and microscopy techniques (Yera et al. 2007).

*Acanthamoeba* as a genus of facultative human parasites is currently classified into 17 genotypes (T1–T17), each of which arguably represents a species (Stothard et al. 1998; Horn et al. 1999; Gast 2001; Hewett et al. 2003; Nuprasert et al. 2010; Corsaro and Venditti 2010).To date, isolates belonging to seven genotypes (T2, T3, T4, T5, T6, T11, and T15) have been found to be associated with AK (Stothard et al. 1998; Booton et al. 2005; Maghsood et al. 2005; Spanakos et al. 2006; Sharifi et al. 2010; Risler et al. 2013). One genotype in particular, T4, was found to be overrepresented in human disease. The main finding of our study is that in Poland, AK is almost always associated with the T4 genotype.

The correlation between *Acanthamoeba* genotype and clinical presentation of AK or susceptibility of the parasite to treatment has not been well explored. Khan (2003) reported that isolates belonging to AK-related genotypes exhibited significantly higher binding and cytotoxicity to corneal epithelial cells than nonclinical isolates. AK is an uncommon diagnosis in Poland. The increased number of positive cases observed in our laboratory may reflect the growing at-risk population of contact lens wearers. The present study was performed on a low number of isolates and may not reflect the entire spectrum of *Acanthamoeba* genotypes occurring in patients in Poland. However, our results indicate that the distribution of genotypes does not differ from that reported from other countries, with T4 as the predominant finding in AK cases.

## References

[CR1] Aniśko-Słomińska J, Słomiński M, Lipowski P (2011). Mikroskopia konfokalna rogówki. Mag Lek Okul.

[CR2] Booton GC, Visvesvara GS, Byers TJ, Kelly DJ, Fuerst PA (2005). Identification and distribution of *Acanthamoeba* species genotypes associated with nonkeratitis infections. J Clin Microbiol.

[CR3] Borin S, Feldman I, Ken-Dror S, Briscoe D (2013). Rapid diagnosis of *Acanthamoeb*a keratitis using non-nutrient agar with a lawn of *E. coli*. J Ophthalmic Inflamm Infect.

[CR4] Caumo K, Frasson AP, Pens CJ, Panatieri LF, Frazzon AP, Rott MB (2009). Potentially pathogenic Acanthamoeba in swimming polls: in survey in the southern Brazilian city of Porto Alegre. Ann Trop Med Parasitol.

[CR5] Corsaro D, Venditti D (2010). Phylogenetic evidence for a new genotype of *Acanthamoeba* (Amoebozoa, Acanthamoebida). Parasitol Res.

[CR6] Corsaro D, Feroldi V, Saucedo G, Ribas F, Loret JF, Greub G (2009). Novel *Chlamydiales* strains isolated from a water treatment plant. Environ Microbiol.

[CR7] Dart JK, Saw VP, Kilvington S (2009). Acanthamoeba keratitis: diagnosis and treatment update 2009. Am J Ophthalmol.

[CR8] Derda M, Wojtkowiak-Giera A, Hadaś E (2014). PCR analysis for the detection of *Acanthamoeba* spp. isolated from water samples. Acta Parasitol.

[CR9] Duarte JL, Furst C, Klisiowicz DR, Klassen G, Costa AO (2013). Morphological, genotypic, and physiological characterization of *Acanthamoeba* isolates from keratitis patients and the domestic environment in Vitoria, Espírito Santo, Brazil. Exp Parasitol.

[CR10] Edagawa A, Kimura A, Kawabuchi-Kurata T, Kusuhara Y, Karanis P (2009). Isolation and genotyping of potentially pathogenic *Acanthamoeba* and *Naegleria* species from tap-water sources in Osaka, Japan. Parasitol Res.

[CR11] Garcia A, Goñi P, Cieloszyk J, Fernandez MT, Calvo-Beguería L, Rubio E, Fillat MF, Peleato ML, Clavel A (2013). Identification of free-living amoebae and amoeba-associated bacteria from reservoirs and water treatment plants by molecular techniques. Environ Sci Technol.

[CR12] Gast RJ (2001). Development of an Acanthamoeba-specific reverse dot-blot and the discovery of a new ribotype. J Eukaryot Microbiol.

[CR13] Gianinazzi C, Schild M, Wuthrich F, Ben Nouir N, Fuchslin HP, Schurch N, Gottstein B, Muller N (2009). Screening Swiss water bodies for potentially pathogenic free-living amoebae. Res Microbiol.

[CR14] Goldschmidt P, Degorge S, Benallaoua D, Batellier L, Di Cave D, Chaumeil C (2012). Rapid detection and simultaneous molecular profile characterization of *Acanthamoeba* infections. Diagn Microbiol Infect Dis.

[CR15] Hewett MK, Robinson BS, Monis PT, Saint CP (2003). Identification of a new *Acanthamoeba* 18S rRNA gene sequence type, corresponding to the species *Acanthamoeba jacobsi* Sawyer, Nerad and Visvesvara, 1992 (Lobosea: Acanthamoebidae). Acta Protozool.

[CR16] Horn M, Fritsche TR, Gautom RK, Schleifer KH, Wagner M (1999). Novel bacterial endosymbionts of *Acanthamoeba* spp. related to the *Paramecium caudatum* symbiont *Caedibacter caryophilus*. Environ Microbiol.

[CR17] Howe DK, Vodkin MH, Novak RJ, Visvesvara G, McLaughlin GL (1997). Identification of two genetic markers that distinguish pathogenic and nonpathogenic strains of *Acanthamoeba* spp. Parasitol Res.

[CR18] Itahashi M, Higaki S, Fukuda M, Mishima H, Shimomura Y (2011). Utility of real-time polymerase chain reaction in diagnosing and treating Acanthamoeba keratitis. Cornea.

[CR19] Jain R, Tilak V (2011). Primary amoebic meningoencephalitis due to *Naegleria fowleri*. J Indian Med Assoc.

[CR20] John DT, Howard MJ (1995). Seasonal distribution of pathogenic free-living amebae in Oklahoma waters. Parasitol Res.

[CR21] Kasprzak W, Mazur T (1972). Free-living amoeba isolated from waters frequented by people in the vicinity of Poznań, Poland. Experimental studies in mice on the pathogenicity of the isolates. Z Tropenmed Parasitol.

[CR22] Khan NA (2003). Pathogenesis of *Acanthamoeba* infections. Microb Pathog.

[CR23] Kokot J, Dobrowolski D, Lyssek-Boroń A, Milka M, Smędowski A, Wójcik Ł, Wowra B, Wylęgała E (2012). Nowe podejście do diagnostyki i leczenia zapaleń rogówki wywołanych przez *Acanthamoeba* sp. –. Klin Ocz.

[CR24] Kosik-Bogacka D, Czepita D, Łanocha N (2010). Pełzaki z rodzaju *Acanthamoeba* jako czynnik etiologiczny zapalenia rogówki oka. Klin Ocz.

[CR25] Laummaunwai P, Ruangjirachuporn W, Boonmars T (2012). A simple PCR condition for detection of a single cyst of *Acanthamoeba* species. Parasitol Res.

[CR26] Le Calvez T, Trouilhe MC, Humeau P, Moletta-Denat M, Frere J, Hechard Y (2012). Detection of free-living amoebae by using multiplex quantitative PCR. Mol Cell Probes.

[CR27] Lehmann OJ, Green SM, Morlet N, Kilvington S, Keys MF, Matheson MM, Dart JK, McGill JI, Watt PJ (1998) Polymerase chain reaction analysis of corneal epithelial and tear samples in the diagnosis of Acanthamoeba keratitis. Invest Ophthalmol Vis Sci 39:1261–1265 9620088

[CR28] Mafra CS, Carrijo-Carvalho LC, Chudzinski-Tavassi AM, Taguchi FM, Foronda AS, Carvalho FR, de Freitas D (2013). Antimicrobial action of biguanides on the viability of Acanthamoeba cysts and assessment of cell toxicity. Invest Ophthalmol Vis Sci.

[CR29] Maghsood AH, Sissons J, Rezaian M, Nolder D, Warhurst D, Khan NA (2005). Acanthamoeba genotype T4 from the UK and Iran and isolation of the T2 genotype from clinical isolates. J Med Microbiol.

[CR30] Magnet A, Galvan AL, Feney S, Izquierdo F, Rueda C, Fernandez VC, Perez-Irezabal J, Bandyopadhyay K, Visvesvara GS, da Silva AJ, del Aquila C (2012). Molecular characterization of Acanthamoeba isolated in water treatment plants and comparison with clinical isolates. Parasitol Res.

[CR31] Mahmoudi MR, Taghipour N, Eftekhar M, Haghighi A, Karanis P (2012). Isolation of *Acanthamoeba* species in surface waters of Gilan province-north of Iran. Parasitol Res.

[CR32] Marciano-Cabral F, Cabral G (2003). *Acanthamoeba* spp. as agents of disease in humans. Clin Microbiol Rev.

[CR33] Naginton J, Watson PG, Playfair TJ, McGill J, Jones BR, Steele AD (1974). Amoebic infection of the eye. Lancet.

[CR34] Nagyova V, Nagy A, Janecek S, Timko J (2010). Morphological, physiological, molecular and phylogenetic characterization of new environmental isolates of *Acanthamoeba* spp. from the region of Bratislava, Slovakia. Biologia.

[CR35] Nagyova V, Nagy A, Timko J (2010). Morphological, physiological and molecular biological characterisation of isolates from first cases of *Acanthamoeba* keratitis in Slovakia. Parasitol Res.

[CR36] Nicholas KB, Nicholas HB Jr. (1997) GeneDoc: a tool for editing and annotating multiple sequence alignments. Distributed by the author (www.cris.com/ketchup/genedoc.shtml)

[CR37] Nicholas KB, Nicholas HB, Deerfield DWI (1997). GeneDoc: analysis and visualization of genetic variation. EMBNETnews.

[CR38] Nuprasert W, Putaporntip C, Pariyakanok L, Jongwutiwes S (2010). Identification of a novel T17 genotype of *Acanthamoeba* from environmental isolates and T10 genotype causing keratitis in Thailand. J Clin Microbiol.

[CR39] Risler A, Coupat-Goutaland B, Pélandakis M (2013). Genotyping and phylogenetic analysis of *Acanthamoeba* isolates associated with keratitis. Parasitol Res.

[CR40] Schroeder JM, Booton GC, Hay J, Niszl IA, Seal DV, Markus MB, Fuerst PA, Byers TJ (2001). Use of subgenic 18S ribosomal DNA PCR and sequencing for genus and genotype identification of Acanthamoebae from humans with keratitis and from sewage sludge. J Clin Microbiol.

[CR41] Seal DV (2003). *Acanthamoeba* keratitis update-incidence, molecular epidemiology and new drugs for treatment. Eye.

[CR42] Sharifi N, Botero-Kleiven S, Ohman D, Barragan A, Winiecka-Krusnell J (2010). Genotypic characterization of *Acanthamoeba* spp. causing ocular infections in Swedish patients: Identification of the T15 genotype in a case of protracted keratitis. Scand J Infect Dis.

[CR43] Spanakos G, Tzanetou K, Miltsakakis D, Patsoula E (2006). Genotyping of pathogenic Acanthamoebae isolated from clinical samples in Greece—report of a clinical isolate presenting T5 genotype. Parasitol Int.

[CR44] Stockman LJ, Wright CJ, Visvesvara GS, Fields BS, Beach MJ (2011). Prevalence of *Acanthamoeba* spp. and other free-living amoebae in household water, Ohio, USA -1990-1992. Parasitol Res.

[CR45] Stothard DR, Schroeder-Diedrich JM, Awwad MH, Gast RJ, Ledee DR, Rodriguez-Zaragoza S, Dean CL, Fuerst PA, Byers TJ (1998). The evolutionary history of the genus Acanthamoeba and the identification of eight new 18 s rRNA gene sequence types. J Eukaryot Microbiol.

[CR46] Thomas V, Loret JF, Jousset M, Greub G (2008). Biodiversity of amoebae and amoebae-resisting bacteria in a drinking water treatment plant. Environ Microbiol.

[CR47] Tsetkova N, Schild M, Panaiotov S, Kurdova-Mintcheva R, Gottstein B, Walochnik J, Aspock H, Lucas MS, Muller N (2004). The identification of free-living environmental isolates of amoebae from Bulgaria. Parasitol Res.

[CR48] Visvesvara GS, Shoff ME, Sriram R, Booton GC, Crary M, Fuerst PA, Hanley CS, Garner MM (2010). Isolation, morphologic, serologic and molecular identification of *Acanthamoeba* T4 genotype from the liver of a Temminck's tragopan (Tragopan temminckii). Vet Parasitol.

[CR49] Yera H, Zamfir O, Bourcier T, Ancelle T, Batellier L, Dupouy-Camet J, Chaumeil C (2007). Comparison of PCR, microscopic examination and culture for the early diagnosis and characterization of *Acanthamoeba* isolates from ocular infections. Eur J Clin Microbiol Infect Dis.

[CR50] Yera H, Zamfir O, Bourcier T, Viscogliosi E, Noël C, Dupouy-Camet J, Chaumeil C (2008). The genotypic characterisation of *Acanthamoeba* isolates from human ocular samples. Br J Ophthalmol.

[CR51] Yoder JS, Verani J, Heidman N, Hoppe-Bauer J, Alfonso EC, Miller D, Jones DB, Bruckner D, Langston R, Jeng BH, Joslin CE, Tu E, Colby K, Vetter E, Ritterband D, Mathers W, Kowalski RP, Acharya NR, Limaye AP, Leiter C, Roy S, Lorick S, Roberts J, Beach MJ (2012). *Acanthamoeba* keratitis: the persistence of cases following a multistate outbreak. Ophthalmic Epidemiol.

